# Global Estimate of Human Brucellosis Incidence

**DOI:** 10.3201/eid2909.230052

**Published:** 2023-09

**Authors:** Christopher G. Laine, Valen E. Johnson, H. Morgan Scott, Angela M. Arenas-Gamboa

**Affiliations:** Texas A&M University, College Station, Texas, USA

**Keywords:** brucellosis, human brucellosis, *Brucella*, brucellosis incidence, brucellosis epidemiology, bacteria

## Abstract

Brucellosis is a major public health concern worldwide, especially for persons living in resource-limited settings. Historically, an evidence-based estimate of the global annual incidence of human cases has been elusive. We used international public health data to fill this information gap through application of risk metrics to worldwide and regional at-risk populations. We performed estimations using 3 statistical models (weighted average interpolation, bootstrap resampling, and Bayesian inference) and considered missing information. An evidence-based conservative estimate of the annual global incidence is 2.1 million, significantly higher than was previously assumed. Our models indicate Africa and Asia sustain most of the global risk and cases, although areas within the Americas and Europe remain of concern. This study reveals that disease risk and incidence are higher than previously suggested and lie mainly within resource-limited settings. Clarification of both misdiagnosis and underdiagnosis is required because those factors will amplify case estimates.

Brucellosis is a bacterial disease that affects populations of livestock and humans, as well as their respective economies, throughout the world (*1*–*4*). Three of the *Brucella* species are highly virulent to their natural hosts, as well as to humans, and are considered endemic in most countries, predominantly in resource-limited settings (*1*,*2*,*4*,*5*). Those species are *B. abortus*, which primarily infects cattle; *B. melitensis*, which infects sheep and goats; and *B. suis*, which infects mainly swine (*4*). Of interest, although *Brucella* infections are a considerable concern for livestock and are known to be zoonotic, human brucellosis is less recognized and understood (*1*,*4*). In humans, the disease is typically characterized by nonspecific influenza-like illness manifesting as undulating fever, sweats, fatigue, and malaise, which are similar signs and symptoms to those of malaria, one of the most commonly acquired infectious diseases in resource-limited regions (*1*,*2*,*4*). Furthermore, undulant fever, arthritis, myocarditis, and neuropathies can occur among chronic cases of human brucellosis (*1*,*4*). Humans are normally exposed to *Brucella* spp. by consuming unpasteurized milk products or handling contaminated tissues such as aborted livestock placentas (*4*). Those exposure pathways put raw milk–product consumers, livestock owners, abattoir workers, and veterinarians at high risk of acquiring the disease within endemic areas (*4*).

Despite the established recognition of the zoonotic risk worldwide (*2*,*6*,*7*), the number of new human brucellosis cases annually remains unclear (*8*). For decades, researchers have attempted to identify the global and regional impact of this disease. However, all previous efforts to quantify the annual number of new cases either have not been based on sufficient, documented evidence (*9*) or have concluded that it was not possible to accurately determine the global incidence of this disease using results available from the scientific literature (*10*,*11*). In addition, annual incidence cannot be estimated solely from human brucellosis cases reported to intergovernmental public health institutions because of incomplete data and lack of representation among geographic regions (*8*).

To enhance understanding of the disease’s effects worldwide, we aimed to identify at-risk human populations worldwide, estimate the risk for populations for which there are currently no available data, estimate the risk of acquiring human brucellosis both globally and regionally, and estimate annual incidence. We produced these estimates using animal and human brucellosis data reported to the World Organization of Animal Health (WOAH, formerly OIE) and human population data reported to the World Bank. To the best of our knowledge, the use of this approach has not previously been attempted. To accomplish these goals, we used 3 data sources: reported animal data that indicates the presence of *B. abortus*, *B. melitensis*, and *B. suis* among the 182 WOAH member states; reported human data compiled by WOAH demonstrating the presence of human brucellosis by country, without regard for *Brucella* species; and rural human population counts within these countries (those with the highest likelihood of contact with livestock) from the World Bank. We used 3 distinct statistical approaches, weighted average interpolation, bootstrap resampling, and Bayesian hierarchical modeling, to estimate incidence and assess the confidence of our results. Our findings suggest that the severity and magnitude of global human brucellosis incidence have been significantly underestimated.

## Materials and Methods

Although precise estimates of the annual incidence of human brucellosis cannot be obtained using existing data repositories or scientific reports alone (*8*,*10*,*11*), this study combines existing data sources and analyses from 3 statistical models to provide estimates of the annual incidence rates and characterize the uncertainty of these estimates. The data used in our analyses represent a combination of open-source data provided by WOAH, showing the presence of disease in animals and human case counts, and by the World Bank, showing national human populations and percentage of rural human populations. The statistical models we propose range from simple weighted average interpolation, to bootstrap resampling, and finally to Bayesian hierarchical models. We also estimate the risk by geographic region (Appendix Figure). We define population risk (i.e., incidence proportion) as the ratio of new cases within a population relative to the total population at risk. Consequently, the number of new cases could be calculated by multiplying the total population at risk by the population risk. This relationship between incidence, the population at risk, and population risk served as the basic framework for all statistical models. Because of the sharp decline in available information during the COVID-19 pandemic (*8*), we used data from the most recent uninterrupted 5-year timeframe (i.e., 2014–2018) (Appendix). To ensure best reporting practices, we conducted the study under the Guidelines for Accurate and Transparent Health Estimates Reporting (GATHER) (*12*).

### General Modeling Procedures

To estimate the baseline human brucellosis incidence data at country and regional levels, we followed the methods described in Laine et al. (*8*). We first stratified the global human population into mutually exclusive country and region groups. To provide a population scale for individual countries, we added the World Bank estimates for each year (2014–2018) (*13*,*14*) into the dataset. Second, to provide a means of geographic comparison, we grouped the individual WOAH member countries into 4 continental regions (Africa, Americas, Asia, or Europe), as specified by WOAH (*5*). We excluded Oceania because its 7 countries and 132 total reported case counts (RCCs) during 2014–2018 provided insufficient data to statistically estimate case counts; those countries have small populations relative to the rest of the world, so they do not substantially affect the overall results. Subsequently, we categorized differences in reporting methods by each country into a mutually exclusive group (e.g., informative versus uninformative) on the basis of the information presented. Informative reports specified a quantified RCC within the report (RCC >0). Uninformative reports provided no quantified information on the human brucellosis status of the country. Because we assessed a 5-year timeframe and not every country reported annually, we took the average RCC as the input parameter from each of the countries that reported >3 of 5 years.

We used our observed RCC input parameters to estimate case counts for the uninformative reports, providing values for the overall regional and global incidence estimates. Specifically, to calculate the overall incidence, we divided the RCC input parameters by their respective populations at risk for the country-level risk (Appendix Figure); this parameter is essential for estimating among each of the models. Country-level risk is equivalent to incidence proportion, which can simply be referred to as risk. We applied this risk, through 3 models (Appendix), to estimate risk for those countries that did not provide RCCs for the study timeframe. After we used each model to estimate the risk for nonreporting countries, we multiplied the risk against each of the respective populations at risk to estimate incidence.

One of the most important risk factors for acquiring brucellosis is close contact with infected livestock, especially by engagement in activities known to increase the risk for infection, such as consuming raw milk and handling infected tissues (*4*,*15*,*16*). Of interest, we found no evidence in previous studies to suggest a certain livestock-to-human ratio as a risk factor. Furthermore, brucellosis is known to be routinely maintained and transmitted in transhumant herds, and wild animals and can propagate in areas with sparse livestock populations (*17*–*20*). What matters for transmission is the probability of contact, driven by the infected to susceptible ratio (routine sustained contact between infectious livestock or products and susceptible humans is more likely on smallholder farms in rural settings) (*17*). The degree of infection in the human population is, therefore, representative of the amount of interaction between infected animals or products and susceptible humans. Worldwide, most livestock reside in rural areas where it is common practice to consume raw milk; therefore, we used the World Bank dataset identifying the percentage of each country’s population that resides in rural areas and multiplied it by the total population of each country to calculate the population at risk for each country (Appendix Figure). We segregated at-risk populations at the country level into different categories: rural populations in every country where brucellosis was reported in humans, rural populations in every country that reported the disease in livestock but that had not submitted RCCs, and rural populations in every country that did not report RCCs or the absence of *Brucella* spp. in livestock (Appendix).

## Results

Previous studies have indicated that an accurate global disease incidence estimation is not possible using reported human data (*8*). Therefore, we used a novel approach to estimate disease incidence along with the uncertainties of those estimates. Our estimates used both human and animal information to identify human at-risk populations worldwide, estimate risk for populations for which there is currently no available data, estimate the risk of acquiring human brucellosis globally and regionally, and estimate annual global and regional incidence.

### Determination of At-Risk Human Populations

**Figure 1:**
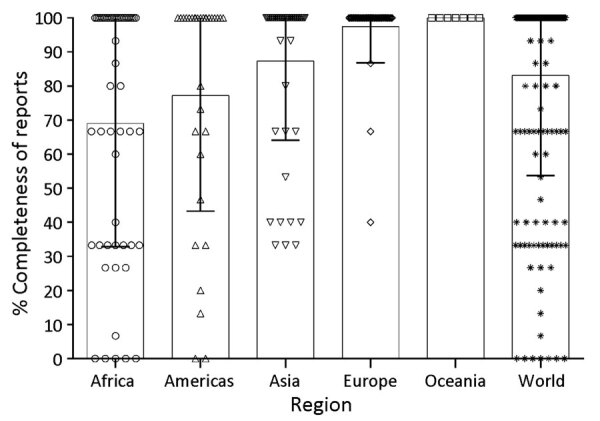
Percentage completeness of World Organization of Animal Health annual reports that provide information on each of the zoonotic *Brucella* species, by worldwide region, 2014–2018. Each point on the plot denotes the 5-year average percentage completeness of reports from an individual country. Reporting the presence or absence of all *Brucella* species (*B. abortus*, *B. melitensis*, and *B. suis*) equates to 100%. Bar tops indicate mean % completeness for each region and error bars indicate SDs from each mean.

Analysis of the livestock datasets indicates that during 2014–2018 a total of 83.1% (2,269/2,730) (population SD 29.4%) of the livestock brucellosis data were provided for the 3 *Brucella* species (Figure 1), compared to 48.4% of human brucellosis data (*8*). Specifically, from the lowest to the highest percentage of reports, Africa provided 69.1% of the expected information on *Brucella* spp. (549/795, SD 36.2%), the Americas 77.2% (359/465, SD 33.9%), Asia 87.3% (642/735, SD 23.2%), and Europe 97.5% (614/630, SD 10.6%) (Figure 1). Because we had more complete data for livestock than human disease at both the global and regional levels, we used livestock data as the basis to estimate disease incidence. Even so, a limiting factor in using the livestock data was the incompleteness of *B. suis,* 76.5% (696/910, SD 40.0%) data compared with *B. melitensis*, 81.4% (741/910, SD 35.5%) and *B. abortus,* 91.4% (832/910, SD 24.4%) data. That information is unavailable for human disease, which further supports our decision to base our analyses on livestock data to identify which *Brucella* species is afflicting each population.

Worldwide, 82.3% (144/175) of countries and 43.2% (3.2 billion/7.4 billion) of persons were considered at risk. By region, 92.5% (49/53) of the countries and 57.5% (0.69 billion/1.2 billion) of persons in Africa, 85.7% (42/49) of countries and 47.7% (2.1 billion/4.4 billion) of persons in Asia, 80.6% (25/31) of countries and 19.4% (0.19 billion/0.98 billion) of persons in the Americas, and 66.7% (28/42) of countries and 24.3% (0.18/0.74 billion) of persons in Europe were at risk. As noted, the model included only 175/182 countries; all of the countries from Oceania were excluded because of incomplete reporting, the small number of countries (7 total) and at-risk population numbers (7.6 million) involved, and the small number of RCCs (132 RCCs over 5 years).

### Determining the Risk of Acquiring Human Brucellosis

**Figure 2:**
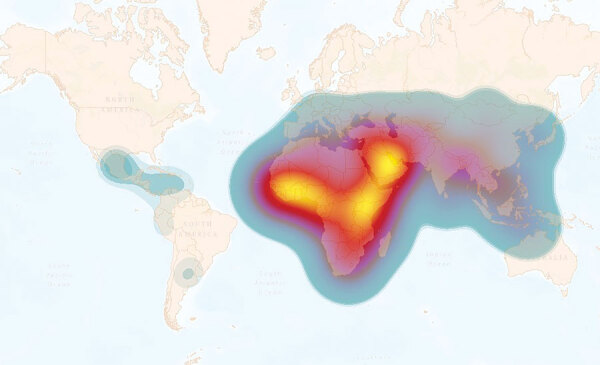
Heat map of global annual incidence of human brucellosis estimated per 1 million population at risk. Overall global risk is defined by the weighted average interpolation data (total number of new cases/total population at risk × 1 million). The global average is ≈500 new cases per 1 million persons at risk. The heat scale shows high risk to low risk; yellow (>4,000 cases) to blue (<1 case). This heatmap is intended to represent transnational zones that require priority control or surveillance initiative, not to represent the risk for individual countries.

Identifying the human populations that are most at risk of acquiring brucellosis is pivotal for the design and implementation of interventions to mitigate disease spread. Therefore, we used the information from countries that reported human disease to calculate the level of risk for their populations at risk. We entered generated data into ArcMap (Esri, https://www.arcgis.com) to produce heat maps. The global average risk was ≈500 new cases/1 million persons (Figure 2). As expected, the maps demonstrate distinct epidemiologic differences between the regions; Africa reflects most of the risk, followed by Asia, then the rest of the world.

### Estimating Annual Incidence

**Figure 3:**
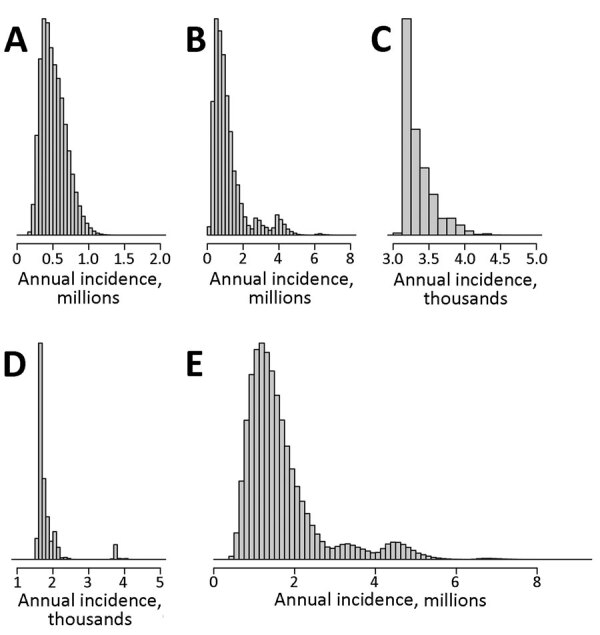
Estimated distribution of annual human brucellosis incidence as determined by bootstrap resampling model for Africa (A), Asia (B), Americas (C), and Europe (D) and globally (E). Histograms generated via 1 million sample iterations based on observed reported case count values.

**Figure 4:**
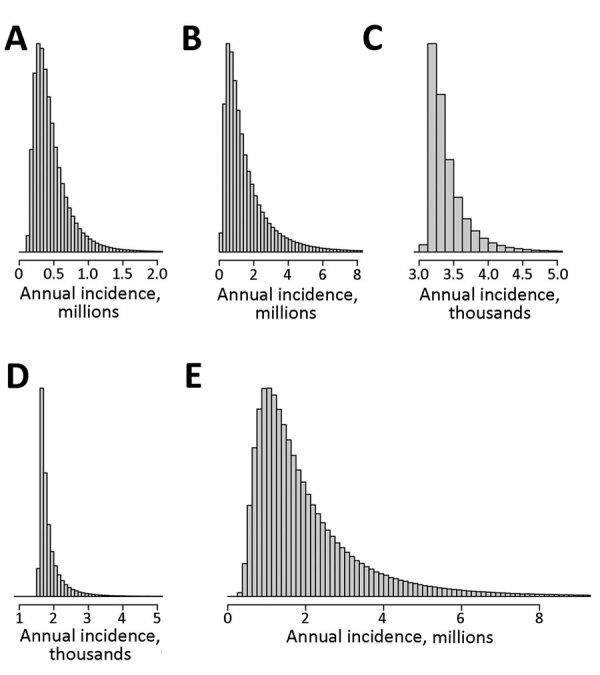
Estimated distribution of annual human brucellosis incidence as determined by Bayesian hierarchical model for Africa (A), Asia (B), Americas (C), and Europe (D) and globally (E). Histograms generated via 1 million sample iterations. Posterior distributions were estimated using a Markov chain Monte Carlo (MCMC) algorithm based on observed reported case count values. For the MCMC algorithm, 50,000 burn-in iterations were performed before the samples were retained.

After population risk assessment, we used 3 models to determine annual incidence. By weighted average interpolation model, the estimated incidence was 1,621,468; by bootstrap resampling model, the mean estimated incidence was 1,691,666; and by Bayesian hierarchical model, the mean estimated incidence was 2,096,080 (Table). Of interest, the models computed similar results between the means and medians both regionally and globally (Table), suggesting some robustness in each approach despite the individual strengths and weaknesses of each. The conservative global annual incidence was 1.6–2.1 million new cases across models. When we analyzed the data by region, Asia (1.2–1.6 million cases) and Africa (0.5 million cases) accounted for most of the cases. Nonetheless, there were also a substantial number of cases in the Americas and Europe. Differences in the results between models were mainly between the smoothness of the resampling histograms (Figure 3, Figure 4) and the distribution of CIs (Table) produced by the bootstrap resampling and hierarchical Bayes frameworks. All models indicated that the global annual incidence of human brucellosis is many times larger than previously thought (*9*).

**Table:** Estimated annual incidence of human brucellosis worldwide determined by using 3 statistical models*

Region	Estimated human cases		2.5% Quantile	25% Quantile	Median	75% Quantile	97.5% Quantile
	Total	Mean (SD)					
Weighted average interpolation							
World	1,621,468						
Asia	1,103,122						
Africa	514,001						
Americas	3,335						
Europe	1,010						
Bootstrap resampling							
World		1,691,666 (975,292)	679,393	1,080,049	1,416,482	1,906,564	4,651,474
Asia		1,172,573 (959,859)	261,493	566,081	887,126	1,355,607	4,107,355
Africa		513,928 (171,607)	257,863	380,681	487,549	624,155	902,139
Americas		3,343 (214)	3,133	3,181	3,272	3,448	3,912
Europe		1,821 (424)	1,595	1,632	1,688	1,818	3,717
Hierarchical Bayes†							
World		2,096,080 (1,754,315)	568,038	1,063,620	1,592,291	2,511,881	6,616,334
Asia		1,622,446 (1,680,985)	246,536	639,906	1,117,309	1,993,573	5,972,342
Africa		468,321 (291,337)	168,919	283,125	393,384	562,957	1,210,226
Americas		3,425 (362)	3,133	3,215	3,319	3,503	4,347
Europe		1,889 (446)	1,593	1,654	1,746	1,944	3,050

*Calculation of uncertainty intervals in the weighted average interpolation method was not performed due to the nature of the model. During bootstrap resampling, uncertainty intervals were calculated using one million resampled risk estimates based on observed reported case count values. †The hierarchical Bayes model intervals were calculated using 1 million posterior samples. Posterior distributions were estimated using a Markov chain Monte Carlo (MCMC) algorithm based on observed reported case count values. For the MCMC algorithm, 50,000 burn-in iterations were performed before the samples were retained.

Using the observed information provided from Europe, the region with the strongest surveillance systems and most complete reports for both humans and livestock, we determined each model’s accuracy in representing the behavior of the system. We estimated 1,010 new cases by weighted average interpolation model, 1,821 cases by bootstrap resampling, and 1,889 cases by hierarchical Bayes model annually in Europe. The average value of empirical annual RCCs from Europe provided by WOAH was 1,771 cases/year; range was 727–5,329. Together with similar results between the means and medians of the models, our findings support both internal and external model validity.

### Overall Regional Risk Assessment

**Figure 5:**
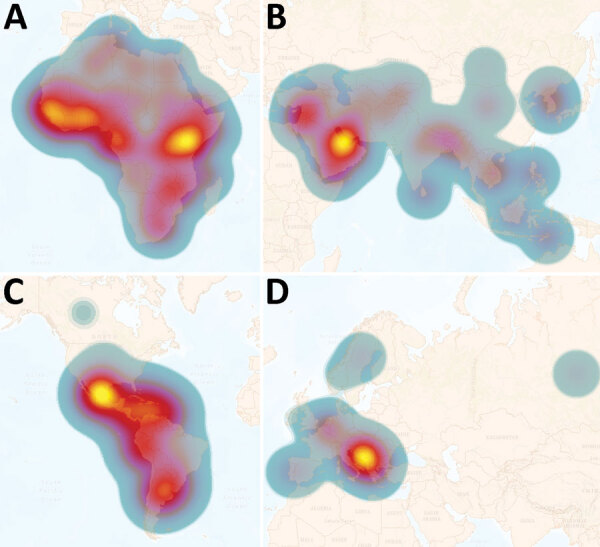
Heatmaps of regional annual incidence of human brucellosis estimated per 1 million population at risk. Each region has a different scale for incidence per 1 million population at risk. Heatmaps are intended to represent transnational zones that require priority control or surveillance initiative, not to represent the risk of individual countries. The heat scale shows high risk to low risk; yellow to blue. A) Africa: average risk is ≈750 new cases per million; high is >3,000. B) Asia: average risk is ≈500 new cases per million; high is >4,000. C) Americas: average risk is ≈20 new cases per million; high is >75. D) Europe: average risk is ≈10 new cases per million; high >100.

We assessed the overall risk at the regional level of acquiring human brucellosis using the incidence and population at risk data and subsequently applying this information to generate heat maps for a visual interpretation of regional risk. As we expected, all regions analyzed in this study have some degree of disease risk, which is primarily focused within the tropics. However, the magnitude of the risk differed substantially among and within regions (Figure 5). Africa is at most significant risk (Figure 5, panel A), followed by Asia (Figure 5, panel B), the Americas (Figure 5, panel C), and Europe (Figure 5, panel D). Within Africa (Figure 5, panel A), all but 4 countries are considered high risk, and 3 of those countries are island nations. Major hotspots occur in the equatorial regions of the east and west, followed by the southern region, and the northern Saharan subregion. In Asia, the major risk hotspot is localized in the Middle East subregion (Figure 5, panel B). With the exception of 6 countries, 5 of which are island nations, all countries in Asia are considered to be at risk, and risk levels are increased in the central, south, and southeast subregions. Although the Americas (Figure 5, panel C) experience less risk, there is more significant subregional variation than in Asia and Africa. Central America experiences most human brucellosis risk. South America follows, having major hotspots in the northern and southern portions of the continent. North America experiences the least risk in this region. Finally, Europe (Figure 5, panel D) is considered to have the least risk of all the analyzed regions, having a major hotspot in the Eastern Mediterranean area and increased risk in the central subregion.

## Discussion

This study provides an empirically based estimate of human brucellosis incidence and associated risk for persons worldwide, suggesting a reality that at least 1.6–2.1 million new cases of human brucellosis likely occur every year. This estimate differs significantly from one of the most cited references in the brucellosis field (*9*), which predicts an incidence of 500,000 new cases yearly. Although that previous estimate was not rigorously justified using empirical data, the estimate of 500,000 new cases has been assumed and used worldwide as a key factor for determining the disease’s global significance and effect on humans. The continued use of that estimate can be attributed mainly to the paucity of data presented by the international reporting system and a lack of empirical evidence that caused the scientific community to ignore the burden of this disease (*8*). As a solution, in this study, we used human and animal data and a range of statistical methods to provide a better understanding of global brucellosis incidence.

It is essential to highlight that we did not incorporate disease misdiagnosis and underdiagnosis into our statistical models as parameters because of limited data. If we had, brucellosis estimates would have been even higher. In areas to which malaria and brucellosis are endemic, recent scientific data indicate that 21%–50% of human brucellosis cases were initially misdiagnosed as malaria, and 4%–11% of the total cases initially diagnosed as malaria were later identified as brucellosis (*14*,*15*). In 1 study, 51% of brucellosis cases were initially misdiagnosed as typhoid fever or pneumonia, and 13% of the total cases initially diagnosed as those 2 diseases were later identified as brucellosis (*14*). Underdiagnosis can arise from several deficiencies in medical and public health systems. Examples include a lack of diagnostic capacity, a lack of knowledge by diagnosticians, and a lack of awareness of public health practitioners to prioritize the disease. Current data are inadequate to estimate the extent of those problems worldwide. Given the magnitude of the reported malaria and typhoid incidence within brucellosis-endemic zones, incorporating those effects would likely increase the estimated disease incidence by millions of cases per year. Future research into human brucellosis misdiagnosis and underdiagnosis is necessary for further insight into disease burden (Appendix).

The data and analyses we present demonstrate that only a small proportion of the world’s population is not subject to brucellosis disease risk. Most human brucellosis cases come from regions with highly dense at-risk populations (Figure 2, Figure 5). These results should be considered in the context of previous studies, which suggest that far less data were being collected in 2022 than 15 years earlier (*8*). Combined with the continuing increase in the world population, particularly in Africa (*8*), there is substantial evidence that world populations are more at risk now than in the past. When the regions are viewed separately, Asia and Africa account for most of the risk and incidence of human brucellosis (Figure 2, Figure 5). Moreover, among countries in Africa, inadequate or nonexistent public and animal health programs perpetuate the status quo (*7*,*8*,*16*,*21*). This uncontrolled disease situation, accompanied by rapid population growth and increased demand for animal products, provides an unfortunate outlook for the future of brucellosis control across this entire region. Although risk is spread across the entire Asia region, the primary hotspot occurs in the Middle East. This increased risk is likely the result of having close contact with small ruminants and consuming their raw milk products (*22*).

The Americas also have a uniform spread of risk across the region with distinct hotspots. Central America has the highest risk, followed by northern and southern South America. Farming in this region includes cattle, small ruminants, and pigs and routinely includes interaction with their infected tissues and fluids. In addition, countries not endemic for the disease incur cases resulting from travel and from trade of raw milk products across national borders (*23*). 

Europe has the most advanced brucellosis surveillance and control programs. Countries in this region account for the most complete and representative data, along with the lowest RCCs (*8*), translating to the lowest estimated case counts and risk (Table; Figure 2, Figure 5). Although Europe generally is less of a concern than the other regions, hotspots are present in the Mediterranean area; a subset of the population is at risk for traveler’s brucellosis, which probably accounts for the increased risk within the central subregion. The differences in incidence and risk can be seen in the Eastern Mediterranean area. Similar to the case for the Americas, countries in Europe that are not endemic for the disease also incur cases related to factors such as travel, laboratory-acquired infections, and trade of raw milk products across national borders (*23*). Fortunately, in Europe, the medical infrastructure is adept in identifying and reporting cases to integrated surveillance networks. Because of the high level of completeness within that data, each of the 3 model estimates are close to the reported account, further supporting the model validity.

In conclusion, although the true annual incidence of human brucellosis remains elusive, we have compiled an evidence-based, scientifically computed estimate. This study reveals that the contemporary disease risk conditions most likely translate to an approximate global annual incidence that is many times higher than what has been previously suggested (i.e., conservatively 1.6–2.1 million). Furthermore, the risk of acquiring the disease was highest within resource-limited regions. It is critical that research be conducted to understand the role of misdiagnosis and underdiagnosis of human brucellosis, because those factors will undoubtably amplify case estimates and risk profiles within those regions.

Appendix Additional information about global estimates of human brucellosis incidence.
