# Supplementary material for: Global Estimate of Human Brucellosis Incidence
Source: Emerg Infect Dis. 2023 Sep;29(9):1789–97. doi: 10.3201/eid2909.230052 (PMC10461652; doi:10.3201/eid2909.230052)
Supplement: Appendix — Additional information about global estimates of human brucellosis incidence. [file 23-0052-Techapp-s1.pdf]

Article DOI: http://doi.org/10.3201/eid2909.230052

EID cannot ensure accessibility for supplementary materials supplied by authors. Readers who have difficulty accessing supplementary content should contact the authors for assistance.

Global Estimate of Human Brucellosis Incidence

Appendix

Methods

Data Collection

Human Brucellosis Data

The WOAH is the primary intergovernmental organization that collects and disseminates human and animal brucellosis data, aggregating and reporting new brucellosis cases via an online database called the World Animal Health Information System (WAHIS) (*1*,*2*). Established in 2005, the WAHIS aggregates self-reported information from the WOAH's 182 permanent member countries (*2*). Every year, the WOAH solicits an annual report from each of its member countries. This report nominally includes an update on the presence or absence of each zoonotic *Brucella* spp. in livestock and the total number of new human cases (*2*). As the zoonosis section of the WOAH annual reports has not yet (December 2022) been integrated into the publicly available WAHIS, we contacted the WOAH's World Animal Health Information and Analysis Department for access to the dataset. They provided case counts of newly diagnosed human brucellosis (i.e., annual cumulative incidence) by year, country, and disease status in their annual reports (*2*). It is essential to mention that these reports are not mandatory, and the WOAH does not certify that reported cases are accurately diagnosed. These reports also do not specify which diagnostic tests or clinical criteria were used to diagnose or confirm disease, nor do they specify the *Brucella* spp. infecting humans (e.g., *B. abortus*, *B. melitensis*, and/or *B. suis*) (*2*). These omissions reduce the usefulness of the data for establishing adequate national policies to control the disease. Without knowledge of the diagnostic techniques or the surveillance and reporting systems, we could not confirm the accuracy of the number of newly diagnosed infections in the database. For this reason, we refer to the reported numbers as "reported case counts" (RCC).

Animal Brucellosis Data

One of the main differences between human and animal data availability is that animal data reported by WOAH-WAHIS contains information for each zoonotic species of *Brucella*, and the extent of reporting is more complete than for humans (*2*). However, livestock data are only reported as present or absent (as opposed to an RCC). Similarly, to the human data, the diagnostic methods are not provided by country, resulting in additional uncertainty.

Population Data

To identify at-risk populations, we extracted annual human population counts and rural population percentages for each of the 182 member countries from the World Bank databank (*3*).

Statistical Analysis of Input Parameters

The completeness and representativeness of human RCCs and livestock disease data were subject to statistical and graphical analysis using GraphPad Prism 9. The full methods and analysis of RCCs, found in Laine, et al. 2022 (*4*), were applied to the livestock dataset to assess information completeness.

Specific Modeling Procedures

Three separate models were then employed to estimate the global and annual incidence of human brucellosis. These included weighted average interpolation, bootstrap resampling, and a Bayesian hierarchical model implemented using R software. Heat maps displaying the magnitude of risk worldwide in terms of cases per million at risk were developed and visualized using the geographic information system (GIS) software ArcMap. Country-level information was intentionally omitted from the estimated incidence results as the data can only be interpreted at the global and regional scale for two reasons: 1) in the weighted average interpolation model the exact country specific data are unreliable, because the RCCs used in this model are known to have several types of error (e.g., diagnostic and reporting) resulting in uncertainties. Additionally, the RCCs for countries that do not report a value were based on the average of regional countries that did. 2) The bootstrap and Bayesian hierarchical models imputed parameters from individual countries who reported RCCs to calculate an overall incidence for the region and world.

Weighted Average Interpolation Model

This model was used to: 1) estimate the average risk from the at-risk countries that provided RCCs within a region and was reapplied to the at-risk countries within the same region that did not report data to allow an overall risk assessment for the region, and 2) estimate individual country incidence based upon the determined risk multiplied against the respective populations at risk. The overall estimates were calculated as the sum of the incidence within each regional cohort (regional incidence) and the sum of the regions (global incidence). One of the restraints of this model is that the weighted average interpolation model does not provide confidence intervals due to direct calculation of incidence; therefore, a bootstrap resampling model was also used.

Bootstrap Resampling Model

This model was used to: 1) allow random sampling within a region to address the uncertainty within individual RCCs and 2) incorporate the variation in RCCs among the countries within a regional cohort providing a confidence interval. Specifically, this model randomly resamples risk values from countries that provide a RCC rate within a region and imputes these rates to at-risk countries within the same region that did not report an RCC value. The RCC value for these countries was imputed by multiplying the rate imputed for each non-reporting country by the at-risk population. The overall estimates were calculated as the sum of the incidence within each regional cohort (regional incidence), and the sum of the regions (global incidence). One million iterations of this process were conducted to produce an uncertainty distribution with values for the mean, standard deviation, and median, as well as 2.5%, 25%, 75%, and 97.5% quantiles.

Bayesian Hierarchical Model

This model was used to 1) use prior information within a region to better address variation in data and uncertainty among the RCCs introduced to the weighted average interpolation and Bootstrap models and 2) incorporate the variation in RCCs among the countries within a regional cohort to obtain confidence (or credible) intervals. Within each region r, $r = 1, \ldots, 4$, the incidence rate in country j was denoted by $p(j,r)$ and the number of individuals at risk was denoted by $n(j,r)$. The RCC for a country j and region r that filed a report was denoted by $y(j,r)$ and was assumed to follow a binomial distribution with infection probability $p(j,r)$ and

denominator $n(j,r)$. To predict cases in a country, say j^*, from region r for which case counts were not reported, we assumed that $p(j^*,r)$ was drawn from a β distribution with parameters $(K(r),\pi (r))$, parameterized so that expected value and variance of $p(j^*,r)$ were $\pi(r)$ and $\pi(r)(1-\pi(r))/(K(r) +1)$, respectively. The prior density assumed for $K(r)$ was assumed to be proportional to a standard Cauchy density restricted to the positive real line, and a $Beta(0.00024,1–0.00024)$ prior was assigned to $\pi(r)$. The value of 0.00024 was chosen to match the empirical infection rate across all reporting countries. The Cauchy distribution was chosen as a prior for $K(r)$ due to its heavy tails; the β distribution assigned to the $\pi(r)$ values contained the equivalent of one individual's outcome and so was not highly informative. Because of the relatively small number of reporting countries in Africa, and the similarity of the method-of-moments estimate of $K(r)$ obtained from the Africa and Asia regions, a common value of $K(r)$ was assumed for these two regions. Distinct values of $\pi(r)$ were estimated for each region.

The posterior distributions for $p(j,r)$, $K(r)$, and $\pi(r)$ were estimated using a Markov chain Monte Carlo (MCMC) algorithm (*5*) based on the observed values of $y(j,r)$ in reporting countries. After 50,000 burn-in iterations, one million sampled values were retained from this algorithm for posterior inference. The posterior-predictive distribution for counts in non-reporting countries was estimated by sampling from the predictive distributions on $p(j^*,r)$ given posterior samples of $K(r)$ and $\pi(r)$. Predictive counts for non-reporting countries were then simulated from binomial distributions with parameters $p(j^*,r)$ and denominators equal to their at-risk population. (In simulation code, a Poisson approximation to the binomial distribution was used to improve computational efficiency for large values of $n(j,r)$). Sensitivity analyses showed that nearly identical results were obtained if the prior on $\pi(r)$ was changed to a $Beta(0.5,0.5)$ density and the prior on $K(r)$ was assumed to be proportional to either $1/(K+1)$ (improper) or $1/(K^3+1)$. Like the bootstrap model, results are expressed in terms of mean, standard deviation, and median, as well as 2.5%, 25%, 75%, and 97.5% quantiles.

Model Validation

To determine whether each of the models accurately represents the behavior of the system, the output data from each model was compared to the information submitted by the region with the most complete reports for both humans and livestock (i.e., Europe). For this

validation, the average value estimated by each of the models was compared to the empirical human brucellosis RCC values provided by WOAH.

Discussion

Limitations

It is essential to recognize that the RCCs are unlikely to accurately represent all the case counts owing to several problems in surveillance and reporting systems globally but heavily weighted to resource-limited settings. Other than misdiagnosis, multiple factors make brucellosis diagnostics and surveillance activities complex and challenging (*6–9*). For example, brucellosis can manifest differently between patients and varies between acute and chronic, as well as symptomatic and asymptomatic infection. In many instances, high cost often leads to diagnoses made on presumptive case definitions (*10*,*11*). Furthermore, the diverse range of tests available for laboratory diagnostics have different necessities, including distinctively equipped laboratories and data analysis requirements. Therefore, correctly identifying cases demands well-trained healthcare professionals with the appropriately equipped facilities to properly differentiate between the infectious agents and to identify co-infections (*10*,*12*,*13*). Critically, these considerations can often also lead to underdiagnosis. To curb the effects of underdiagnosis, a significant effort is required to develop local capacity and raise awareness of public health personnel to effectively identify the disease and provide effective treatment.

In addition to problems caused by misdiagnosis and underdiagnosis, many countries fail to report brucellosis incidence data. For example, China routinely provided RCCs to WOAH until 2015 but has not reported them since. Nonetheless, scientific literature suggests that China has comparatively higher incidence rates (*5*,*14*). India lacks national reporting, even though scientific reports suggest high numbers of cases (*15*). Taken together, these countries constitute large proportions of Asian and World populations, and the information not being provided could potentially increase estimated annual case counts regionally and globally.

Overall, we have identified information gaps (*16*), used innovative epidemiologic methods to fill the gaps, and provided evidence. Incorporating this new information with new evidence on the effectiveness of interventions (*17*) should lead to a better path in the future. Yet, as with any modeling paradigm, there are limitations. First, the nonparametric bootstrap may not

reflect all uncertainty in the data because it does not account for the binomial variation in the rates and unknown variation between regions. Additionally, there are not enough data to reasonably estimate the rates in Oceania, and the available data would seem to be unrepresentative because of the missing data (i.e., Australia and New Zealand are the only reporting countries, and they are unlikely to be representative of the others in the region due to their comparative population sizes). Furthermore, our model identifies only rural residents as at-risk populations. Although urban populations can also be at risk from contact with small ruminants, consumption of purchased raw milk and milk products, and workers at slaughter facilities, the burden is primarily centered on rural inhabitants. Moreover, these estimates are very conservative due to underreporting cases, even in the countries that regularly report.

This conservative estimation can be appreciated during resampling within the bootstrap and Bayesian paradigms, with a far right-hand skew of the resulting histograms (Figures 3, 4) being most likely due to the lack of surveillance rigor worldwide. Unfortunately, it appears that countries incurring the majority of the disease burden also have the weakest surveillance and control programs (*16*). This results in a shift of the estimates toward the underreporting countries. Therefore, the primary limitation with estimating the incidence of human brucellosis is the reliability of the input parameters, especially since human data are sparse, animal data are vague, and mis- and under-diagnosis information is nebulous. In the future, data robustness can be remedied but will require a concerted effort among members of the international community. Countries like Kenya have shown that prioritization and reallocation of resources toward surveillance and control have provided better information (*16*,*18*,*19*).

References

1. World Health Organization. Brucellosis. In: Fact sheets. 2020 [cited 2023 Jul 24]. https://www.who.int/news-room/fact-sheets/detail/brucellosis

2. World Organisation for Animal Health. World animal health information system. 2023 [cited 2023 Jul 24]. https://wahis.oie.int

3. World Bank Group. Rural population (% of total population). 2021 [cited 2023 Jul 24]. https://data.worldbank.org/indicator/SP.RUR.TOTL.ZS

4. Laine CG, Scott HM, Arenas-Gamboa AM. Human brucellosis: widespread information deficiency hinders an understanding of global disease frequency. PLoS Negl Trop Dis. 2022;16:e0010404. PubMed https://doi.org/10.1371/journal.pntd.0010404

5. Zhao C, Yang Y, Wu S, Wu W, Xue H, An K, et al. Search trends and prediction of human brucellosis using Baidu index data from 2011 to 2018 in China. Sci Rep. 2020;10:5896. PubMed https://doi.org/10.1038/s41598-020-62517-7

6. Godfroid J, Al Dahouk S, Pappas G, Roth F, Matope G, Muma J, et al. A "One Health" surveillance and control of brucellosis in developing countries: moving away from improvisation. Comp Immunol Microbiol Infect Dis. 2013;36:241–8. PubMed https://doi.org/10.1016/j.cimid.2012.09.001

7. Robinson R. Emergency prevention system for transboundary animal and plant pests and diseases. Guidelines for coordinated human and animal brucellosis surveillance. Rome, Italy: Food and Agriculture Organization of the United Nations; 2003.

8. Khan MZ, Zahoor M. An overview of brucellosis in cattle and humans, and its serological and molecular diagnosis in control strategies. Trop Med Infect Dis. 2018;3:65. PubMed https://doi.org/10.3390/tropicalmed3020065

9. Ibironke AA, McCrindle CM, Fasina FO, Godfroid J. Evaluation of problems and possible solutions linked to the surveillance and control of bovine brucellosis in sub-Saharan Africa, with special emphasis on Nigeria. Vet Ital. 2008;44:549–56. PubMed

10. Franco MP, Mulder M, Gilman RH, Smits HL. Human brucellosis. Lancet Infect Dis. 2007;7:775–86. PubMed https://doi.org/10.1016/S1473-3099(07)70286-4

11. Corbel MJ. Brucellosis in humans and animals. Geneva: World Health Organization; 2006.

12. Njeru J, Melzer F, Wareth G, El-Adawy H, Henning K, Pletz MW, et al. Human brucellosis in febrile patients seeking treatment at remote hospitals, northeastern Kenya, 2014–2015. Emerg Infect Dis. 2016;22:2160–4. PubMed https://doi.org/10.3201/eid2212.160285

13. Bouley AJ, Biggs HM, Stoddard RA, Morrissey AB, Bartlett JA, Afwamba IA, et al. Brucellosis among hospitalized febrile patients in northern Tanzania. Am J Trop Med Hyg. 2012;87:1105–11. PubMed https://doi.org/10.4269/ajtmh.2012.12-0327

14. Zheng R, Xie S, Lu X, Sun L, Zhou Y, Zhang Y, et al. A systematic review and meta-analysis of epidemiology and clinical manifestations of human brucellosis in China. BioMed Res Int. 2018;2018:5712920. PubMed https://doi.org/10.1155/2018/5712920

15. Singh BB, Khatkar MS, Aulakh RS, Gill JPS, Dhand NK. Estimation of the health and economic burden of human brucellosis in India. Prev Vet Med. 2018;154:148–55. PubMed https://doi.org/10.1016/j.prevetmed.2018.03.023

16. Laine CG, Scott HM, Arenas-Gamboa AM. Human brucellosis: widespread information deficiency hinders an understanding of global disease frequency. PLoS Negl Trop Dis. 2022;16:e0010404. PubMed https://doi.org/10.1371/journal.pntd.0010404

17. Nuvey FS, Arkoazi J, Hattendorf J, Mensah GI, Addo KK, Fink G, et al. Effectiveness and profitability of preventive veterinary interventions in controlling infectious diseases of ruminant livestock in sub-Saharan Africa: a scoping review. BMC Vet Res. 2022;18:332. PubMed https://doi.org/10.1186/s12917-022-03428-9

18. Mbabu M, Njeru I, File S, Osoro E, Kiambi S, Bitek A, et al. Establishing a One Health office in Kenya. Pan Afr Med J. 2014;19:106. PubMed https://doi.org/10.11604/pamj.2014.19.106.4588

19. Munyua P, Bitek A, Osoro E, Pieracci EG, Muema J, Mwatondo A, et al. Prioritization of zoonotic diseases in Kenya, 2015. PLoS One. 2016;11:e0161576. PubMed https://doi.org/10.1371/journal.pone.0161576

20. Gilks WR, Richardson S, Spiegelhalter DJ. Markov Chain Monte Carlo in practice. London: Chapman & Hall; 1996.

Appendix Figure (following pages). Analytical flowchart for the estimation of annual human brucellosis incidence. Ovals represent input data, squared boxes represent computed intermediate data, rounded boxes represent modeling processes, and starred boxes represent intermediate and final results. The flowchart is color-coded by model component: general processes applied to all models, in black; weighted average interpolation, in red; bootstrap resampling, in blue; hierarchical Bayes model, in green. MCMC, Markov chain Monte Carlo; RCC, reported case count.

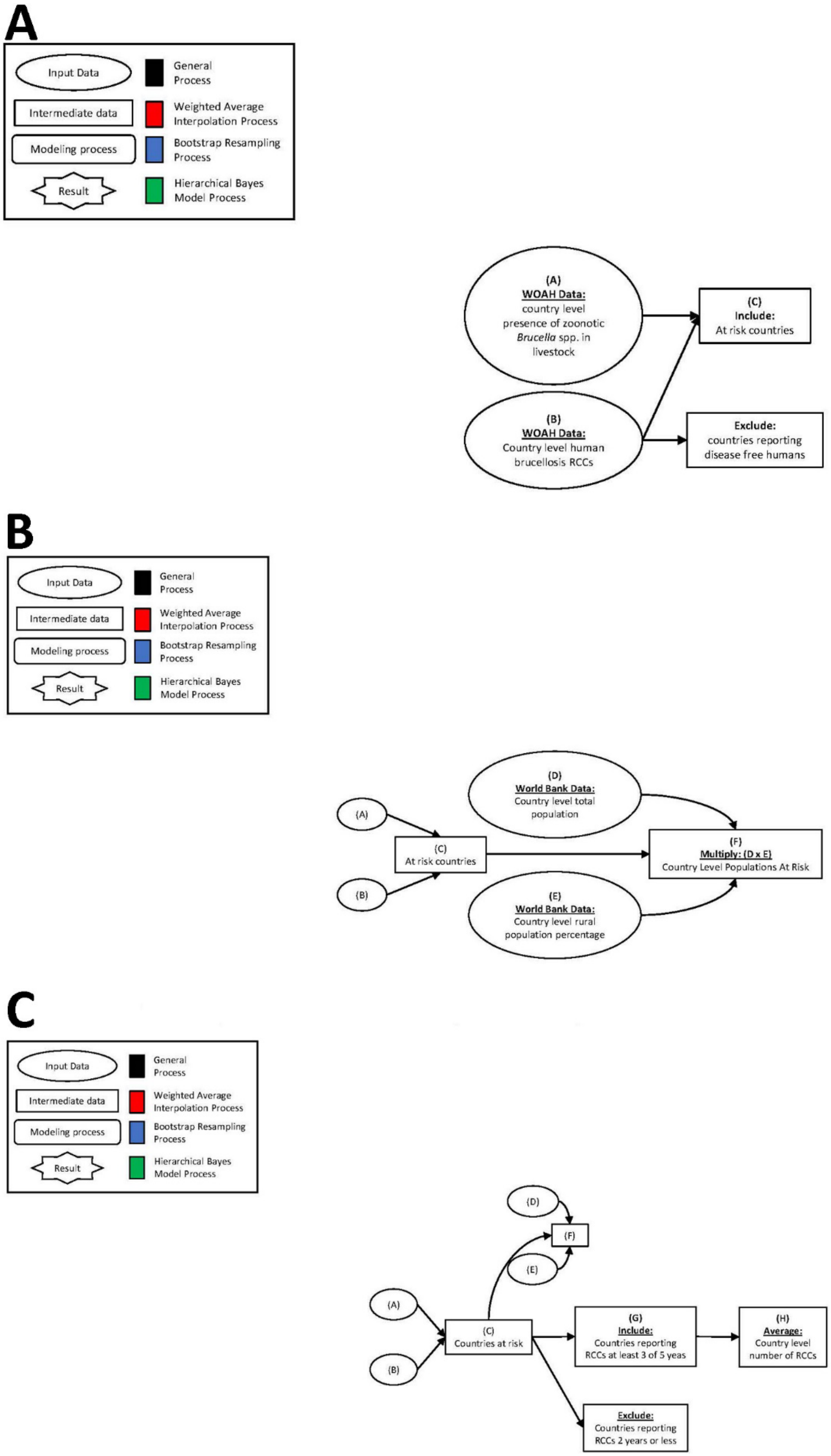
A
Input Data
General Process
Intermediate data
Weighted Average Interpolation Process
Modeling process
Bootstrap Resampling Process
Result
Hierarchical Bayes Model Process
(A) WOAH Data: country level presence of zoonotic Brucella spp. in livestock
(B) WOAH Data: Country level human brucellosis RCCs
(C) Include: At risk countries
Exclude: countries reporting disease free humans
B
Input Data
General Process
Intermediate data
Weighted Average Interpolation Process
Modeling process
Bootstrap Resampling Process
Result
Hierarchical Bayes Model Process
(A)
(B)
(C) At risk countries
(D) World Bank Data: Country level total population
(E) World Bank Data: Country level rural population percentage
(F) Multiply: (D x E) Country Level Populations At Risk
C
Input Data
General Process
Intermediate data
Weighted Average Interpolation Process
Modeling process
Bootstrap Resampling Process
Result
Hierarchical Bayes Model Process
(D)
(F)
(E)
(A)
(B)
(C) Countries at risk
(G) Include: Countries reporting RCCs at least 3 of 5 yeas
(H) Average: Country level number of RCCs
Exclude: Countries reporting RCCs 2 years or less

D

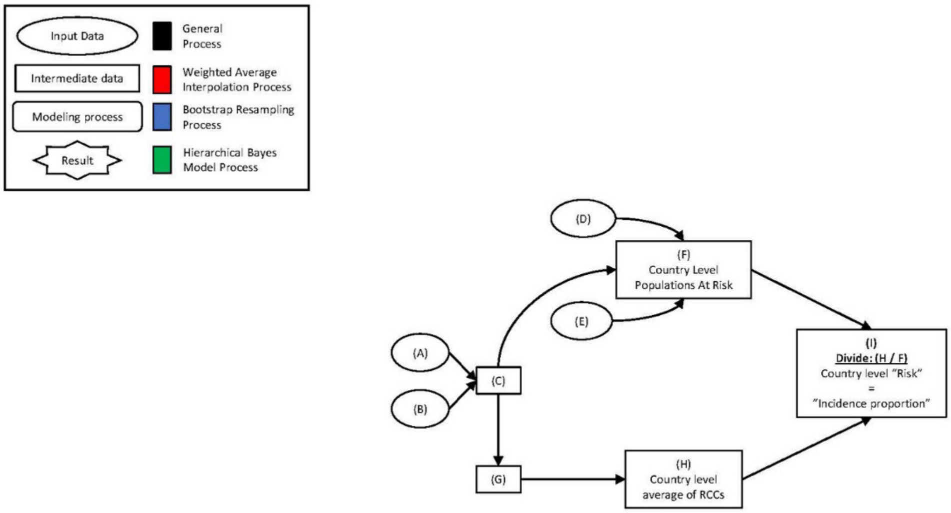

Input Data
General Process
Intermediate data
Weighted Average Interpolation Process
Modeling process
Bootstrap Resampling Process
Result
Hierarchical Bayes Model Process
(D)
(F) Country Level Populations At Risk
(E)
(A)
(B)
(C)
(I) Divide: (H / F) Country level "Risk" = "Incidence proportion"
(G)
(H) Country level average of RCCs

E

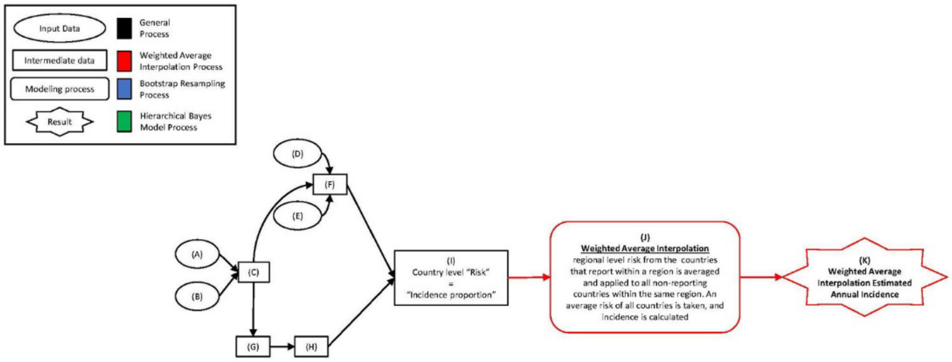

Input Data
General Process
Intermediate data
Weighted Average Interpolation Process
Modeling process
Bootstrap Resampling Process
Result
Hierarchical Bayes Model Process
(D)
(F)
(E)
(A)
(B)
(C)
(G)
(H)
(I) Country level "Risk" = "Incidence proportion"
(J) Weighted Average Interpolation regional level risk from the countries that report within a region is averaged and applied to all non-reporting countries within the same region. An average risk of all countries is taken, and incidence is calculated
(K) Weighted Average Interpolation Estimated Annual Incidence

F

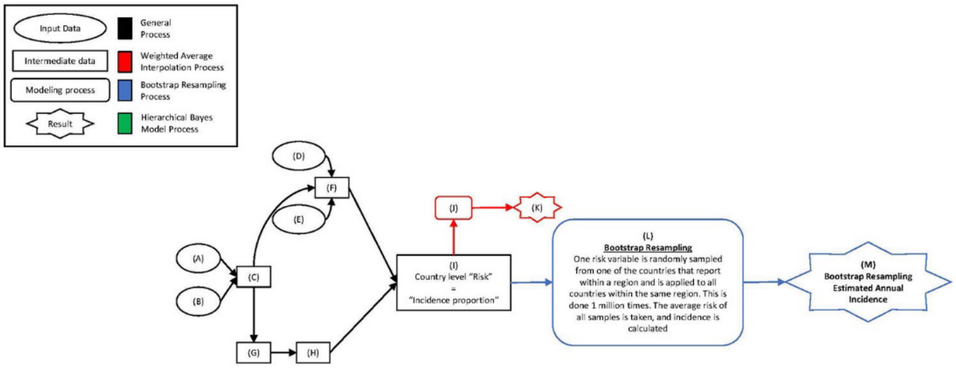

Input Data
General Process
Intermediate data
Weighted Average Interpolation Process
Modeling process
Bootstrap Resampling Process
Result
Hierarchical Bayes Model Process
(D)
(F)
(E)
(A)
(B)
(C)
(G)
(H)
(J)
(K)
(I) Country level "Risk" = "Incidence proportion"
(L) Bootstrap Resampling One risk variable is randomly sampled from one of the countries that report within a region and is applied to all countries within the same region. This is done 1 million times. The average risk of all samples is taken, and incidence is calculated
(M) Bootstrap Resampling Estimated Annual Incidence

G

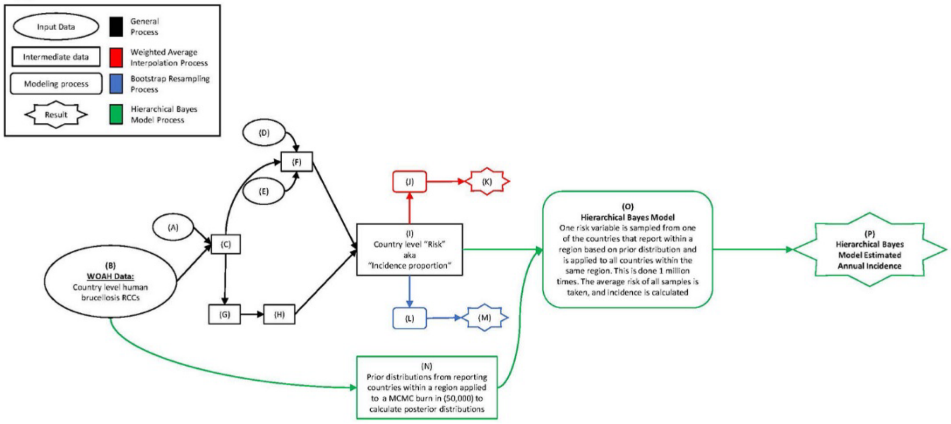

Input Data
General Process
Intermediate data
Weighted Average Interpolation Process
Modeling process
Bootstrap Resampling Process
Result
Hierarchical Bayes Model Process
(A)
(B) WOAH Data: Country level human brucellosis RCCs
(C)
(D)
(E)
(F)
(G)
(H)
(I) Country level "Risk" aka "Incidence proportion"
(J)
(K)
(L)
(M)
(N) Prior distributions from reporting countries within a region applied to a MCMC burn in (50,000) to calculate posterior distributions
(O) Hierarchical Bayes Model One risk variable is sampled from one of the countries that report within a region based on prior distribution and is applied to all countries within the same region. This is done 1 million times. The average risk of all samples is taken, and incidence is calculated
(P) Hierarchical Bayes Model Estimated Annual Incidence

H

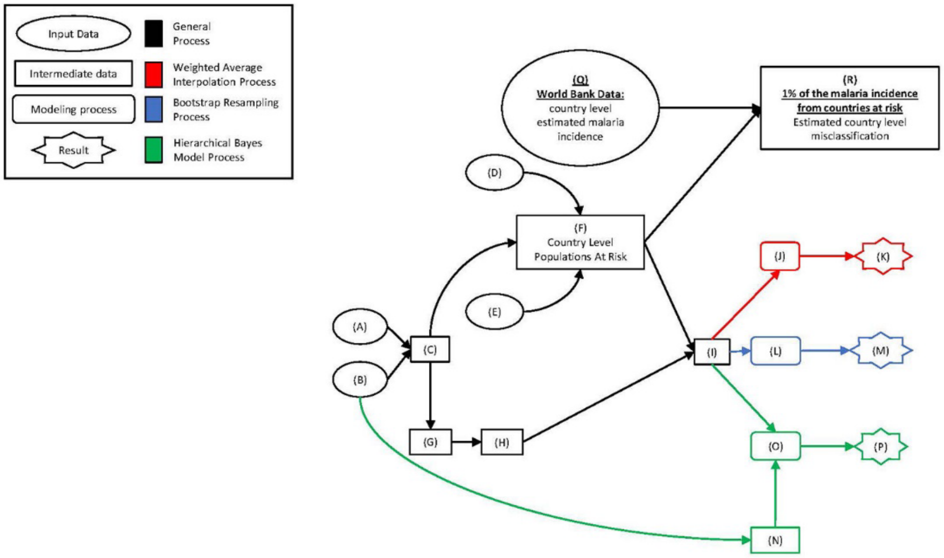

Input Data
General Process
Intermediate data
Weighted Average Interpolation Process
Modeling process
Bootstrap Resampling Process
Result
Hierarchical Bayes Model Process
(Q) World Bank Data: country level estimated malaria incidence
(R) 1% of the malaria incidence from countries at risk Estimated country level misclassification
(D)
(F) Country Level Populations At Risk
(E)
(A)
(B)
(C)
(G)
(H)
(I)
(J)
(K)
(L)
(M)
(O)
(P)
(N)

I

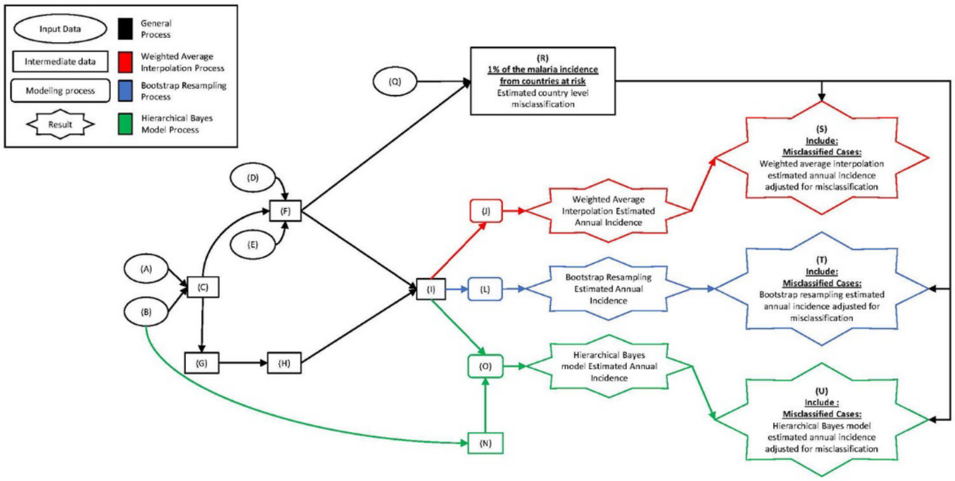

Input Data
General Process
Intermediate data
Weighted Average Interpolation Process
Modeling process
Bootstrap Resampling Process
Result
Hierarchical Bayes Model Process
(Q)
(R) 1% of the malaria incidence from countries at risk Estimated country level misclassification
(S) Include: Misclassified Cases: Weighted average interpolation estimated annual incidence adjusted for misclassification
(D)
(F)
(E)
(J)
Weighted Average Interpolation Estimated Annual Incidence
(A)
(B)
(C)
(I)
(L)
Bootstrap Resampling Estimated Annual Incidence
(T) Include: Misclassified Cases: Bootstrap resampling estimated annual incidence adjusted for misclassification
(G)
(H)
(O)
Hierarchical Bayes model Estimated Annual Incidence
(U) Include : Misclassified Cases: Hierarchical Bayes model estimated annual incidence adjusted for misclassification
(N)
